# Carvedilol alleviates lipopolysaccharide (LPS)-induced acute lung injury by inhibiting Ras homolog family member A (RhoA)/ROCK activities

**DOI:** 10.1080/21655979.2021.2011013

**Published:** 2022-02-21

**Authors:** Jing Xu, Shipin Zhao, Li Zhao, Mengxiu Sun

**Affiliations:** a Department of Respiratory Medicine, Second People’s Hospital of Yueqing, Zhejiang Province, China; b Clinical Laboratory, Second People’s Hospital of Yueqing, Zhejiang Province, China

**Keywords:** Carvedilol, acute lung injury, Rhoa/ROCK

## Abstract

Carvedilol possess multiple functions such as antioxidation and neuroprotection RhoA/ROCK is reported to participate in acute lung injury (ALI). The aim of the present study was to explore the role of carvedilol in LPS-induced ALI. BEAS2B cells were subjected to LPS for the construction of in vitro ALI model. After that, the protective effects of carvedilol were evaluated by Cell Counting Kit-8 (CCK-8). The activities of RhoA/ROCK were then measured to confirm its association with carvedilol by quantitative reverse transcription PCR (RT-qPCR) and Western blot. Then, the cell viability, inflammatory responses, oxidative stress and apoptosis were detected by CCK-8, enzyme linked immunosorbent assay (ELISA), oxidative stress detection kits, and TdT-mediated dUTP Nick-End Labeling (TUNEL) respectively. Inflammation- and apoptosis-related markers were also measured by Western blot. The cell viability reduced by LPS in BEAS2B cells was elevated by carvedilol. Moreover, RhoA/ROCK were found to be suppressed by carvedilol administration. The cell viability, inflammation, oxidative stress and apoptosis of LPS-induced BEAS2B cells were aggravated upon RhoA was overexpressed. Collectively, carvedilol exerts a protective effect against LPS-induced injury that could be ascribed to its anti-inflammatory and antioxidative character through modulating the RhoA/ROCK activities.

## Introduction

Acute lung injury (ALI) is a severe clinical syndrome which causes high morbidity and mortality and is characterized by inflammatory response which the lungs cannot undertake [1]. This disease is clinically manifested with acute onset, progressive hypoxemia and bilateral lung infiltrates on chest radiographs [2,3]. The complicated pathogenesis of ALI has contributed to the difficulty in identifying effective management options for ALI. It is well-known that ALI is triggered as an outcome of G-bacillus infection, by which LPS induces the inflammatory response of lung cells [4]. Thus, we used LPS to induce the in vitro ALI model.

Carvedilol, the third generation of vasodilator with multiple functions such as antioxidation and neuroprotection, is widely used in the treatment of ischemic cardiac disease and hypertension [5]. It is currently used to reduce portal vein pressure and obstruct esophageal and gastric variceal bleeding [6]. Carvedilol has strong anti-inflammatory and antioxidative effects *in vivo* and *in vitro*, but few studies have reported its role in lung injury. Accumulating evidence has suggested that carvedilol can reduce the release of paraquat-induced lung inflammation and oxidative stress, and alleviate the silicosis injury [7,8]. Specifically, the anti-inflammatory and antioxidative effects of carvedilol on alcohol-induced liver injury rats have drawn our attention. RhoA, an essential GTPase protein that modulates cell division, has been associated with the biological processes of cells, including proliferation, differentiation, and migration [9–11]. A study has indicated the effect of carvedilol on reducing the expression of RhoA and ROCK2 in the RhoA/ROCK signaling in hepatic stellate cells induced by angiotensin [12]. Interestingly, inhibition of RhoA/ROCK was demonstrated to alleviate ALI [13]. Emerging evidence show that TLR-4/NF-kappaB pathway is involved in ALI and the modulation of drugs for this pathway inhibits inflammation and apoptosis in improving ALI [14–16]. Carvedilol could play a protective role in improving ALI, which is predicted to be related to RhoA/ROCK signaling. This study aims to explore whether carvedilol can inhibit LPS-induced ALI through RhoA/ROCK signaling, thereby illustrating the potential effects and mechanism of action of carvedilol in ALI.

## Materials and methods

### Cell culture

Human bronchial epithelial cells (BEAS2B, Sigma, Taufkirchen, Germany) were cultured in RPMI-1640 medium (Hyclone, Logan, UT, USA) supplemented with 100 µg/ml streptomycin, 100 U/ml penicillin and 10% fetal bovine serum (Sijiqing, Hangzhou, China) in a humidified incubator with 5% CO_2_ at 37°C. For the following experiments, cells were collected and cultured in 96-well plates with culture medium overnight. BEAS2B cells were treated with different concentration of carvedilol (1, 2.5, 5 and 10 µM) for 24 h. In the cotreatment group of carvedilol and LPS, the cells were pretreated with carvedilol for 1 h and then stimulated with LPS (1 μg/mL) for 6 h.

### Cell transfection

BEAS2B cells were transfected with plasmids overexpressing RhoA (Ov-RhoA, Sangon Biotech, Shanghai, China) or its control plasmids (empty plasmids, Ov-NC) using Lipofectamine® 2000 reagent (Invitrogen; Thermo Fisher Scientific, Inc.) according to the manufacturer’s recommendations. Following 48 h of transfection, the cells were collected for the following experiments.

### CCK-8

The cell viability was assessed using the cell counting kit-8 (CCK-8) assay (Dojindo Laboratories, Kumamoto, Japan). Cell suspension was placed into a 96-well plate (6000 cells per well), which was pre-incubated for 24 h under a humidified atmosphere at 37°C with 5% CO_2_. 10 μL CCK-8 solution was added into each well of the plate. The optical density at 450 nm was monitored using a spectrophotometer.

### RT-qPCR

Total RNA was extracted from transfected BEAS2B cells by the use of TRIzol reagent (Invitrogen; Thermo Fisher Scientific, Inc.). cDNA was synthesized by random hexamer primers presented in the RevertAid First-Strand cDNA synthesis kit (Applied Biosystems; Thermo Fisher Scientific, Inc., Cat no. K1621). The reaction conditions were as follows: 37°C for 15 min and 85°C for 5 sec. qPCR was performed using the SYBR Green Master Mix (Takara, Dalian, China) based on the suggestions provided by the manufacturer. The used primers in this study are as following: RhoA (Sequence (5ʹ -> 3ʹ) Forward: AGCCTGTGGAAAGACATGCTT, Reverse: TCAAACACTGTGGGCACATAC. GAPDH (Sequence (5ʹ -> 3ʹ) Forward: GGAGCGAGATCCCTCCAAAAT, Reverse: GGCTGTTGTCATACTTCTCATGG. Relative mRNA expression was determined using the 2^−ΔΔCq^ method [17], and GAPDH served as an internal control.

### Western blot

BEAS2B cells treated with RIPA lysis buffer, were homogenized for the collection of cell supernatant, and then total proteins were extracted according to the manufacturer’s recommendations. Quantification of proteins was performed by BCA protein quantification kits (ab102536; Abcam). Then, the protein samples were separated by 12% SDS-PAGE gel and transferred to polyvinylidene fluoride membranes. Nonspecific binding was blocked with 5% skim milk in Tris-buffered saline (TBS) at room temperature for 4 h. The membranes were then incubated with the primary antibodies (p-MYPT1, sc-377,542; total-MYPT1, sc-514,261; p-p65, sc-166,748; p-65, sc-8008; 1:1000. SANTA CRUZ BIOTECHNOLOGY, INC. RhoA, ab187027, 1:5000; TLR4, ab13556, 1:500; pCox2, ab179800; Bax, ab32503; Bad, ab32445; cleaved caspase3, ab32042; Bcl-2, ab32124; 1:1000. GAPDH, ab8245, 1:5000, abcam, England) at 4°C overnight, following which was TBS washing for three times and the incubation with an HRP-labeled goat anti-rabbit IgG secondary antibody (1:10,000, abcam, England) at room temperature for 1.5 h. The bands were visualized using an enhanced chemiluminescent detection system (Bio-Rad, Hercules, CA, USA). Band intensities were determined using the ImageJ software (National Institutes of Health, Bethesda, MA, USA).

### ELISA

TNF-α, Interleukin (IL)-1β, and IL-6 quantification was performed using an ELISA Kit (KeyGEN, Nanjing, China) according to the manufacturer’s recommendations. The evaluation of their values was conducted by an iMark microplate reader (Bio-Rad Laboratories, Inc., CA, USA).

### Detection of ROS, MDA and SOD

The content of ROS, malondialdehyde (MDA) and superoxide dismutase (SOD) in cells were monitored using commercial kits (ROS, Reactive oxygen species Assay Kit, E004-1-1; MDA: Malondialdehyde (MDA) assay kit, A003-1-2; SOD: Superoxide Dismutase (SOD) assay kit (WST-1 method), A001-3-2, Nanjing Jiancheng Bioengineering Institute; Nanjing, China), respectively.

### Transferase dUTP nick end labeling (TUNEL) staining

The TUNEL staining was conducted to evaluate cell apoptosis in accordance with the manufacturer’s protocol. For apoptosis of BEAS2B cells, the cells washed by phosphate-buffered saline for twice and fixed by 4% paraformaldehyde. Subsequently, the TUNEL kits (Beyotime, China) were used for staining of the apoptotic cells. The nuclei of healthy cells were stained blue, whereas apoptotic cells with nuclei presented green staining were recognized as TUNEL-positive cells.

### Statistical analysis

The data in this paper were analyzed using GraphPad prism version 6.0 (GraphPad Software, Inc.). Comparisons among multiple groups were conducted by one-way analysis of variance (ANOVA) followed by Tukey’s test. Each experiment was repeated three times at least. A P value of <0.05 represented a statistically significant difference.

## Results

### The effect of carvedilol on cell viability of LPS-induced BEAS2B cells

**Figure 1.**
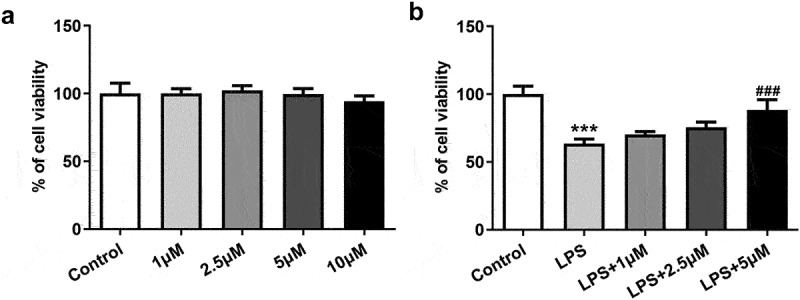
The effect of carvedilol on cell viability of LPS-induced BEAS2B cells. (a) The cell viability of BEAS2B cells treated with carvedilol. (b) The cell viability of LPS-induced BEAS2B cells treated with carvedilol. ***P < 0.001 Versus Control, ^###^P < 0.001 Versus LPS.

To investigate the therapeutic effect of carvedilol on ALI, we first determined if there was any adverse effect of carvedilol on normal BEAS2B cells. After BEAS2B cells were administrated with different doses of carvedilol (1, 2.5, 5 and 10 μM), we found that 10 μM carvedilol did little cytotoxicity on these cells, and thus we abandoned this dosage for the preciseness of the experimental results (Figure 1(a)). After LPS induction and carvedilol administration, the cell viability of BEAS2B cells was again tested. Evidently, the cell viability damaged by LPS was increasingly reverted by carvedilol (Figure 1(b)).

### Carvedilol reduces the expression of RhoA and ROCK in LPS-induced BEAS2B cells

**Figure 2.**
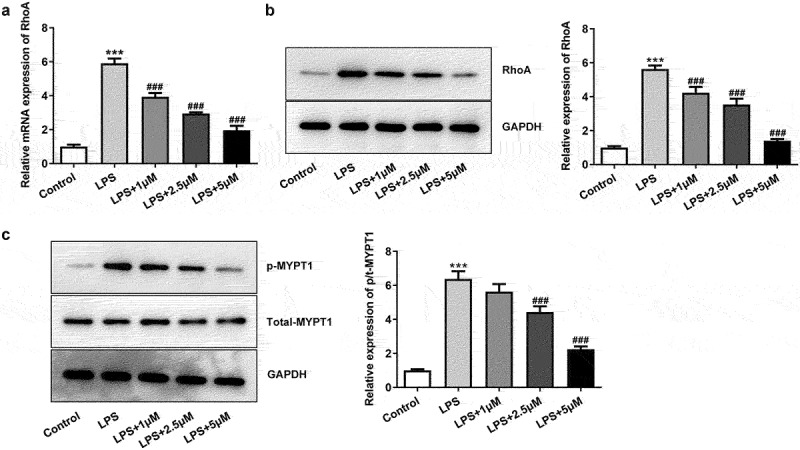
Carvedilol reduces the expression of RhoA and ROCK in LPS-induced BEAS2B cells. (a-b) The expression of RhoA in LPS-induced BEAS2B cells. (c) The expression of MYPT1 in LPS-induced BEAS2B cells. ***P < 0.001 Versus Control, ^###^P < 0.001 Versus LPS.

LPS-induced BEAS2B cells to observe whether carvedilol was involved in the regulation of RhoA/ROCK signaling. As presented inFigure 2(a), the expression of RhoA elevated by LPS was decreased after different doses of carvedilol were added. The ROCK activity could be eanalyzed by the evaluation of MYPT1 phosphorylation levels at Thr696 [18], and thus MYPT1 expression was measured by Western blot. It was obviously seen in Figure 2(b), the MYPT1 was dramatically phosphorylated at a high level, but further administration of carvedilol weakened this effect. Thus, we can conclude that carvedilol reduces the expression of RhoA and ROCK in LPS-induced BEAS2B cells.

### Carvedilol reduces the cell viability, inflammation and oxidative stress of LPS-induced BEAS2B cells by inhibiting RhoA/ROCK activities

**Figure 3.**
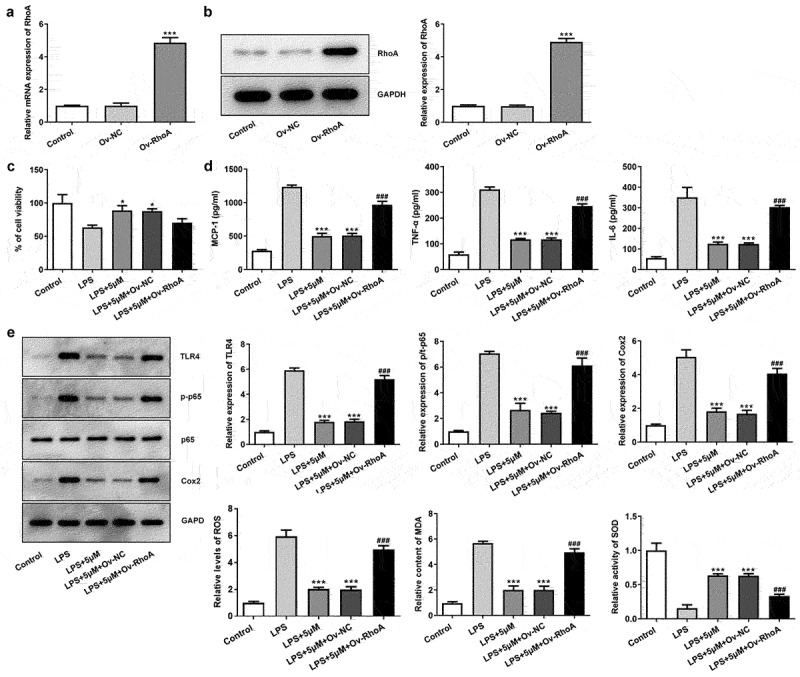
Carvedilol reduces the cell viability, inflammation and oxidative stress of LPS-induced BEAS2B cells by inhibiting RhoA/ROCK activities. (a-b) The expression of RhoA after the plasmid overexpressing RhoA was constructed. ***P < 0.001 Versus Ov-NC. (c) The cell viability of LPS-induced BEAS2B cells transfected with Ov-RhoA. (d) The expression of inflammatory cytokines in LPS-induced BEAS2B cells transfected with Ov-RhoA, detected by ELISA. (e) The expression of inflammation-related markers in LPS-induced BEAS2B cells transfected with Ov-RhoA, detected by Western blot. (f) The levels of ROS, MDA, and SOD in LPS-induced BEAS2B cells transfected with Ov-RhoA *P < 0.05, ***P < 0.001 Versus LPS. ^###^P < 0.001 Versus LPS+5 µM+Ov-NC.

To further investigate the role of RhoA/ROCK in LPS-induced ALI, RhoA was overexpressed and its expression was significantly increased compared with control (Figure 3(a-b)). Intriguingly, when we treated LPS-induced BEAS2B cells with both 5 μm carvedilol and Ov-RhoA, the cell viability was decreased compared with LPS+5 μM group (Figure 3(c)). The alterations in inflammatory response were further explored by determining the production of pro-inflammatory cytokines. ELISA analysis showed that LPS-stimulated elevation of pro-inflammatory cytokine production was weakened by carvedilol, which was abrogated by Ov-RhoA (Figure 3(d)). The present study also demonstrated that indicators of inflammation exhibited higher levels upon LPS challenge, whereas further administration of carvedilol reduced their levels (Figure 3(e)). Nevertheless, Ov-RhoA upregulated their levels. Thus, these data indicated that carvedilol reduces the cell viability and inflammation of LPS-induced BEAS2B cells by inhibiting RhoA/ROCK activities. Following the above assays, oxidative stress and apoptosis of LPS-induced BEAS2B cells were determined after Ov-RhoA was injected into these cells. Decreased levels of ROS and MDA and increased activities of SOD when carvedilol was used on LPS-induced BEAS2B cells were displayed in Figure 3(f).

### Carvedilol reduces apoptosis of LPS-induced BEAS2B cells by inhibiting RhoA/ROCK activities

**Figure 4.**
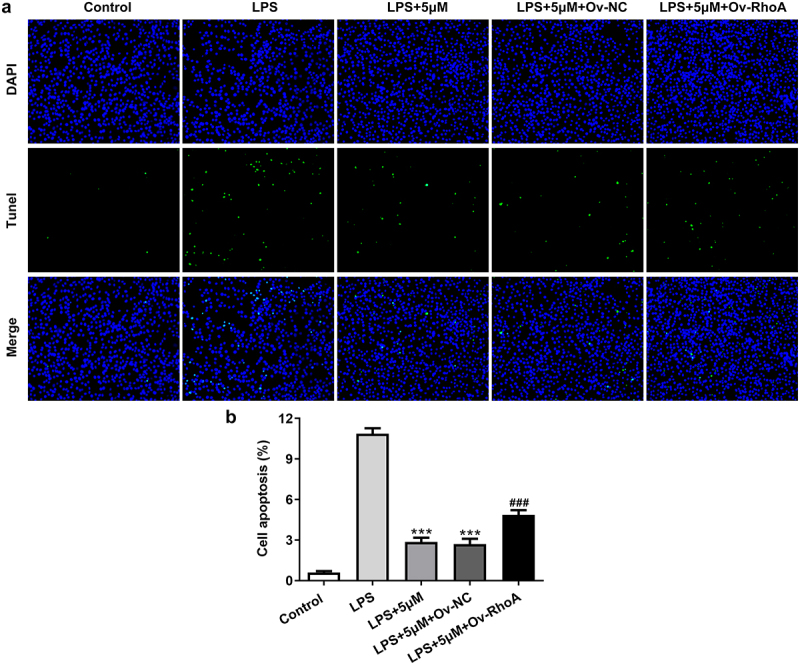
Carvedilol reduces apoptosis of LPS-induced BEAS2B cells by inhibiting RhoA/ROCK activities. (a-b) The apoptosis of LPS-induced BEAS2B cells transfected with Ov-RhoA. ***P < 0.001 Versus LPS. ^###^P < 0.001 Versus LPS+5 µM+Ov-NC.

**Figure5.**
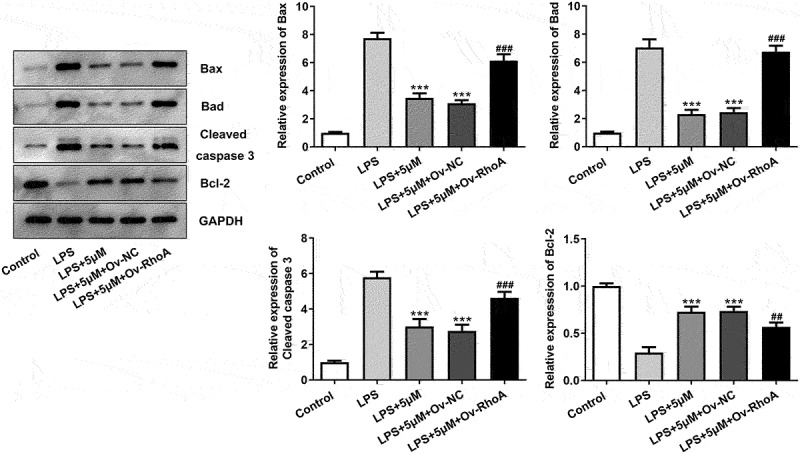
Carvedilol affects the expression levels of apoptosis-related proteins in LPS-induced BEAS2B cells by inhibiting RhoA/ROCK activities. The expression of apoptosis-related proteins, including Bax, Bad, cleaved caspase3 and Bcl-2 in LPS-induced BEAS2B cells transfected with Ov-RhoA. ***P < 0.001 Versus LPS. ^##^P < 0.01, ^###^P < 0.001 Versus LPS+5 µM+Ov-NC.

To evaluate the effects of carvedilol on apoptosis in LPS-challenged BEAS2B cells, the apoptotic levels and the expression of apoptosis-related proteins were detected. As shown in Figures 4 and 5, Ov-RhoA reversed their levels. Results in Figure 4(a) demonstrated by TUNEL suggested that the apoptosis of LPS-induced BEAS2B cells attenuated by carvedilol was again enhanced after RhoA overexpression (Figure 4(b)). LPS stimulation induced increased levels of Bax, Bad and cleaved caspase-3 protein, and decreased Bcl-2 expression levels. Intriguing, carvedilol treatment was able to reduce levels of Bax, Bad and cleaved caspase-3 protein, and increased Bcl-2 expression levels when compared with LPS alone. When the overexpression of RhoA was induced, these effects of carvedilol were markedly reversed (Figure 5). Taken together, these results suggest that carvedilol reduces the oxidative stress and apoptosis of LPS-induced BEAS2B cells by inhibiting RhoA/ROCK activities.

## Discussion

ALI is an inflammatory disorder where inflammatory responses and disruption of the lung endothelial and epithelial barriers occur [19]. Following the in-depth investigations into the pathogenesis of ALI is the research on novel targets and pharmacologic drugs for the reduction of the disease mortality. The attenuation of carvedilol has been previously indicated by several lines of evidence. For instance, carvedilol is able to improve the overall survival rate of cirrhosis patients, presenting the sufferers with an opportunity to improve their health and quality of life [20]. A randomized controlled trial has also reported the improved survival of liver failure patients by carvedilol treatment [21].

In this study, we focused on the effects of carvedilol on LPS-induced ALI and revealed the underlying mechanism. We first determined that low and middle doses (1, 2.5, and 5 μm) of carvedilol did no harm to the viability of BEAS2B cells, and then LPS was used to induce the ALI model in BEAS2B cells. The results in this study indicated that the restoration of cell injury was dependent upon carvedilol administration since the BEAS2B cell viability damaged by LPS was reversed by carvedilol.

RhoA/ROCK signaling pathway, which is able to regulate diverse biological processes, is widely acknowledged to be associated with pathophysiological status in humans [22]. The regulation of this pathway is involved in the treatment of various diseases since abnormal RhoA/ROCK signaling results in the pathophysiology of a large number of disorders, including traumatic brain injury, osteoarthritis and diabetic nephropathy [23,24]. Specifically, a study describing the role of Rho kinase inhibitors has indicated the abnormal high level of RhoA/ROCK signaling pathway leads to inflammation, immune cell migration and apoptosis of pulmonary endothelial cells, which are linked to endothelium barrier dysfunction and edema, hallmarks of ALI [25]. Therefore, we speculated that carvedilol might exert its alleviative effects on LPS-induced via targeting this pathway. Consistent with the abovementioned finding, we found that carvedilol administration downregulated the expression of RhoA and ROCK, as demonstrated by the decreased levels of RhoA and gradually dephosphorylated MYPT1 in LPS-induced BEAS2B cells treated with carvedilol. To further validate the association between carvedilol and RhoA/ROCK signaling, LPS-induced BEAS2B cells pretreated with carvedilol were transfected with Ov-RhoA, and interestingly, the cell viability was decreased as a result.

Notably, the association of inflammation with RhoA/ROCK signaling was paid much attention to herein. Oxymatrine had protective effects on mice with acute intestinal inflammation by obstructing RhoA/ROCK signaling pathway [26]. The inflammation in myocardial ischemia-reperfusion injury rat model was suppressed by Vitamin D via inhibition of RhoA/ROCK signaling pathway [27]. Concurrently, the result in the study that Ov-RhoA stimulated the production of inflammatory cytokines including TNF-α, IL-1β, and IL-6 implicated the suppressive role of carvedilol in ALI via RhoA/ROCK signaling. Oxidative stress and apoptosis were also involved in the pathogenesis of ALI. The current study provided evidence that carvedilol reduced the oxidative stress and apoptosis of LPS-induced BEAS2B cells by inhibiting RhoA/ROCK activities.

## Conclusion

Collectively, carvedilol exerts a potential protective effect on ALI that could be ascribed to its anti-inflammatory and antioxidative character, which regulate the RhoA/ROCK activities. Therefore, carvedilol might be a promising drug for the prevention or management of ALI.

## Data Availability

The analyzed data sets generated during the present study are available from the corresponding author on reasonable request.

## Highlights

1. Carvedilol suppresses RhoA/ROCK signaling in LPS-stimulated human bronchial epithelial cells.

2. Carvedilol exerts potential effects against ALI by decreasing inflammation, apoptosis and oxidative stress.

3. RhoA/ROCK signaling is involved in the protective effects of carvedilol against ALI.
